# Interocular symmetry of optical coherence tomography parameters in healthy children and adolescents

**DOI:** 10.1038/s41598-021-04563-3

**Published:** 2022-01-13

**Authors:** Mi Yeon Song, Young Hoon Hwang

**Affiliations:** 1 Department of Ophthalmology, Kim’s Eye Hospital, Seoul, Republic of Korea; 2 Department of Ophthalmology, Chungnam National University Hospital, 282 Munhwa-ro, Jung-gu, Daejeon, 35015 Republic of Korea

**Keywords:** Neuroscience, Diseases, Medical research, Neurology

## Abstract

Evaluation of interocular asymmetry of optical coherence tomography (OCT) parameters is important for the glaucoma and optic neuropathies. This study was performed to evaluate the interocular asymmetry of OCT parameters in healthy children and adolescents. The circumpapillary retinal nerve fiber layer (RNFL) thickness, optic nerve head (ONH) parameters, and macular ganglion cell-inner plexiform layer (GCIPL) thickness were measured in 620 eyes of 310 healthy children and adolescents using Cirrus HD-OCT. The interocular asymmetry (right eye–left eye) in the OCT parameters was analyzed. The mean ± standard deviation age was 10.3 ± 3.7 years (range 5–17). The right eyes showed thinner superior quadrant RNFL, thicker nasal and temporal quadrant RNFL, lesser rim and disc areas, and thinner average, superior, and superonasal GCIPL than the left eyes (P < 0.05). The 2.5th and 97.5th percentile interocular difference tolerance limits were − 9.0 μm and 11.0 μm for average RNFL thickness, − 0.21 and 0.18 for average cup-to-disc ratio, and − 4.0 μm and 4.0 μm for average GCIPL thickness, respectively. Interocular differences were found in RNFL thickness, ONH parameters, and GCIPL thickness in healthy children and adolescents. These findings should be considered when comparing OCT parameters between the right and left eyes.

## Introduction

It has been reported that interocular asymmetry (difference between the right and left eyes) in circumpapillary retinal nerve fiber layer (RNFL) thickness, optic nerve head (ONH) parameters, and macular retinal ganglion cell (RGC) thickness measured by optical coherence tomography (OCT) may be an early sign of a glaucomatous change^1–3^. Therefore, it would be useful to investigate the normal range and affecting factors of the interocular difference in RNFL thickness, ONH parameters, and RGC thickness.

To date, only a few studies have assessed the interocular symmetry of OCT parameters in children and adolescents^4–10^. In addition to the classical RNFL and ONH evaluation using ophthalmoscope, fundus photographs, or OCT, advancement of OCT technology enabled automatic segmentation of RGC layers. Currently, macular RGC thickness measurement using OCT is essential for the evaluation of glaucoma and other optic neuropathies^11–14^. However, little is known about the interocular symmetry of macular RGC thickness in children and adolescents^10^. Furthermore, no study investigated interocular symmetry of RNFL thickness, ONH parameters, and macular RGC thickness simultaneously. When comparing the results of various studies, differences in participant characteristics were the main confounding factors for direct comparison among the studies. The best way to minimize this effect would be to obtain all possible parameters for the same participants. Therefore, simultaneous analysis of interocular symmetry of RNFL thickness, ONH parameters, and GCIPL thickness with a large number of children and adolescents may provide a useful information for the detection of glaucoma and various optic neuropathies in them. This study was performed to evaluate the interocular symmetry of RNFL thickness, ONH parameters, and RGC thickness in healthy children and adolescents.

## Results

This study included 620 eyes from 310 healthy children and adolescents (150 females, 160 males). All participants were Korean. The mean ± standard deviation age was 10.3 ± 3.7 years (range 5–17) and mean refractive error was − 1.29 ± 2.16 D (range − 7.50 to + 5.50) in the right eye and − 1.37 ± 2.10 D (range − 6.75 to + 5.50) in the left eye. The interocular difference in the refractive error was not significant (P = 0.148).

### The interocular differences in OCT parameters

The interocular differences in OCT parameters between the right and left eyes are presented in Table 1. The mean average RNFL thickness was 102.7 ± 9.3 μm in the right eye and 102.2 ± 9.1 μm in the left eye. The right eyes showed thinner superior quadrant RNFL, thicker nasal and temporal quadrant RNFL (P < 0.001). Among the ONH parameters, the right eyes showed lesser rim (P = 0.001) and disc areas (P = 0.002); cup-to-disc ratio and cup volume did not show significant differences (P > 0.05). Regarding the macular GCIPL thickness, the right eyes had a thinner GCIPL in average, superior, and superonasal sectors compared to the left eyes (P < 0.05).

**Table 1:** Interocular differences (right eye–left eye) in optical coherence tomography parameters between the right and left eyes (n = 310).

	Right eye	Left eye	P value*
**RNFL thickness (µm)**			
Average	102.7 ± 9.3 (79–144)	102.2 ± 9.1 (79–136)	0.070
**Quadrant**			
Superior	128.9 ± 14.6 (90–172)	132.6 ± 16.3 (92–190)	< 0.001
Nasal	70.1 ± 11.7 (43–114)	66.5 ± 10.6 (44–108)	< 0.001
Inferior	131.5 ± 16.7 (86–204)	131.4 ± 16.3 (89–191)	0.948
Temporal	80.6 ± 13.5 (54–164)	77.9 ± 12.7 (49–155)	< 0.001
**ONH parameters**			
Rim area (mm^2^)	1.45 ± 0.27 (0.90–2.35)	1.48 ± 2.62 (0.89–2.48)	0.001
Disc area (mm^2^)	2.02 ± 0.39 (1.08–3.30)	2.06 ± 0.42 (1.22–3.32)	0.002
Average cup-to-disc ratio	0.48 ± 0.17 (0.06–0.75)	0.48 ± 0.17 (0.10–0.80)	0.709
Vertical cup-to-disc ratio	0.44 ± 0.16 (0.05–0.70)	0.43 ± 0.17 (0.05–0.72)	0.271
Cup volume (mm^3^)	0.177 ± 0.161 (0–0.845)	0.177 ± 0.168 (0–1.064)	0.954
**GCIPL thickness (µm)**			
Average	84.8 ± 4.5 (70–98)	85.1 ± 4.6 (70–98)	0.019
Minimum	82.3 ± 4.3 (71–94)	82.5 ± 4.4 (70–96)	0.092
Superotemporal	83.7 ± 4.9 (70–98)	83.8 ± 4.7 (71–96)	0.262
Superior	85.2 ± 4.9 (68–99)	85.8 ± 4.9 (69–104)	< 0.001
Superonasal	86.9 ± 4.6 (70–101)	87.8 ± 5.0 (69–103)	< 0.001
Inferonasal	85.6 ± 5.0 (68–101)	85.3 ± 5.0 (68–102)	0.150
Inferior	82.6 ± 5.2 (67–101)	82.8 ± 5.2 (67–99)	0.620
Inferotemporal	84.8 ± 5.3 (72–100)	84.8 ± 5.1 (71–99)	0.823

Data are presented as mean ± standard deviation (range).

*RNFL* retinal nerve fiber layer, *ONH* optic nerve head, *GCIPL* ganglion cell-inner plexiform layer.

*Paired *t* test.

**Figure 1:**
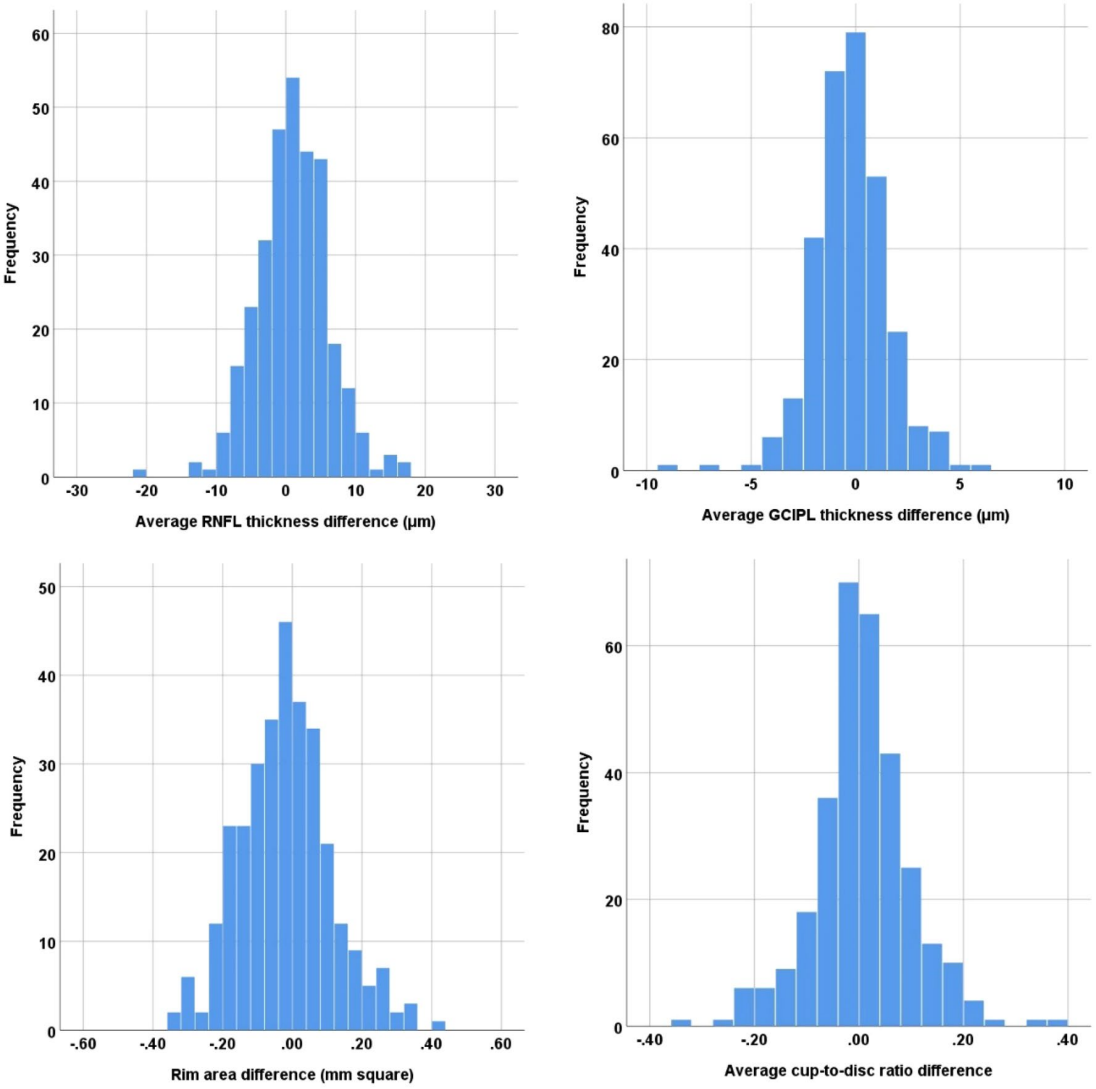
The distribution of interocular difference in the average retinal nerve fiber layer (RNFL) thickness, average ganglion cell-inner plexiform layer (GCIPL) thickness, rim area, and average cup-to-disc ratio between the right and left eyes.

The distribution of interocular difference in the average RNFL thickness, average GCIPL thickness, rim area, and average cup-to-disc ratio between the right and left eyes is presented in Fig. 1. The percentile distribution of the interocular differences in the OCT parameters between the right and left eyes is shown in Table 2. The 2.5th and 97.5th percentile interocular difference tolerance limits were − 9.0 μm and 11.0 μm for the average RNFL thickness, − 0.21 and 0.18 for average cup-to-disc ratio, and − 4.0 μm and 4.0 μm for average GCIPL thickness, respectively.

**Table 2:** Percentile distribution of interocular differences (right eye–left eye) in optical coherence tomography parameters between the right and left eyes (n = 310).

	Mean ± SD (range)	Percentile			
		2.5th	5th	95th	97.5th
**RNFL thickness (µm)**					
Average	0.5 ± 5.1 (− 21.0 to 16.0)	− 9.0	− 8.0	9.0	11.0
**Quadrant**					
Superior	− 3.8 ± 12.1 (− 45.0 to 41.0)	− 29.5	− 25.5	14.5	19.2
Nasal	3.6 ± 8.0 (− 17.0 to 33.0)	− 13.0	− 9.0	17.0	20.0
Inferior	0.0 ± 11.3 (− 40.0 to 31.0)	− 26.5	− 19.0	17.0	20.5
Temporal	2.7 ± 7.9 (− 27.0 to 29.0)	− 14.0	− 9.5	18.0	19.2
**ONH parameters**					
Rim area (mm^2^)	− 0.03 ± 0.13 (− 0.33 to 0.43)	− 0.29	− 0.22	0.21	0.27
Disc area (mm^2^)	− 0.04 ± 0.23 (− 1.01 to 0.76)	− 0.59	− 0.44	0.31	0.36
Average cup-to-disc ratio	0.00 ± 0.09 (− 0.33 to 0.37)	− 0.21	− 0.16	0.16	0.18
Vertical cup-to-disc ratio	0.01 ± 0.09 (− 0.30 to 0.40)	− 0.23	− 0.14	0.16	0.18
Cup volume (mm^3^)	0.000 ± 0.077 (− 0.264 to 0.234)	− 0.185	− 0.144	0.132	0.166
**GCIPL thickness (µm)**					
Average	− 0.2 ± 1.8 (− 9.0 to 6.0)	− 4.0	− 3.0	3.0	4.0
Minimum	− 0.2 ± 2.1 (− 6.0 to 8.0)	− 4.0	− 4.0	4.0	4.2
Superotemporal	− 0.2 ± 2.6 (− 9.0 to 15.0)	− 5.0	− 4.0	3.0	4.0
Superior	− 0.6 ± 2.8 (− 12.0 to 11.0)	− 6.0	− 5.0	4.0	5.0
Superonasal	− 0.9 ± 2.7 (− 12.0 to 10.0)	− 7.0	− 6.0	3.0	4.0
Inferonasal	0.3 ± 3.0 (− 12.0 to 10.0)	− 6.0	− 4.5	4.0	5.2
Inferior	0.1 ± 3.3 (− 13.0 to 10.0)	− 6.0	− 5.0	6.5	8.0
Inferotemporal	0.0 ± 2.8 (− 11.0 to 14.0)	− 6.0	− 4.0	4.0	5.0

*RNFL* retinal nerve fiber layer, *ONH* optic nerve head, *GCIPL* ganglion cell-inner plexiform layer.

### Affecting factors for the interocular differences

**Figure 2:**
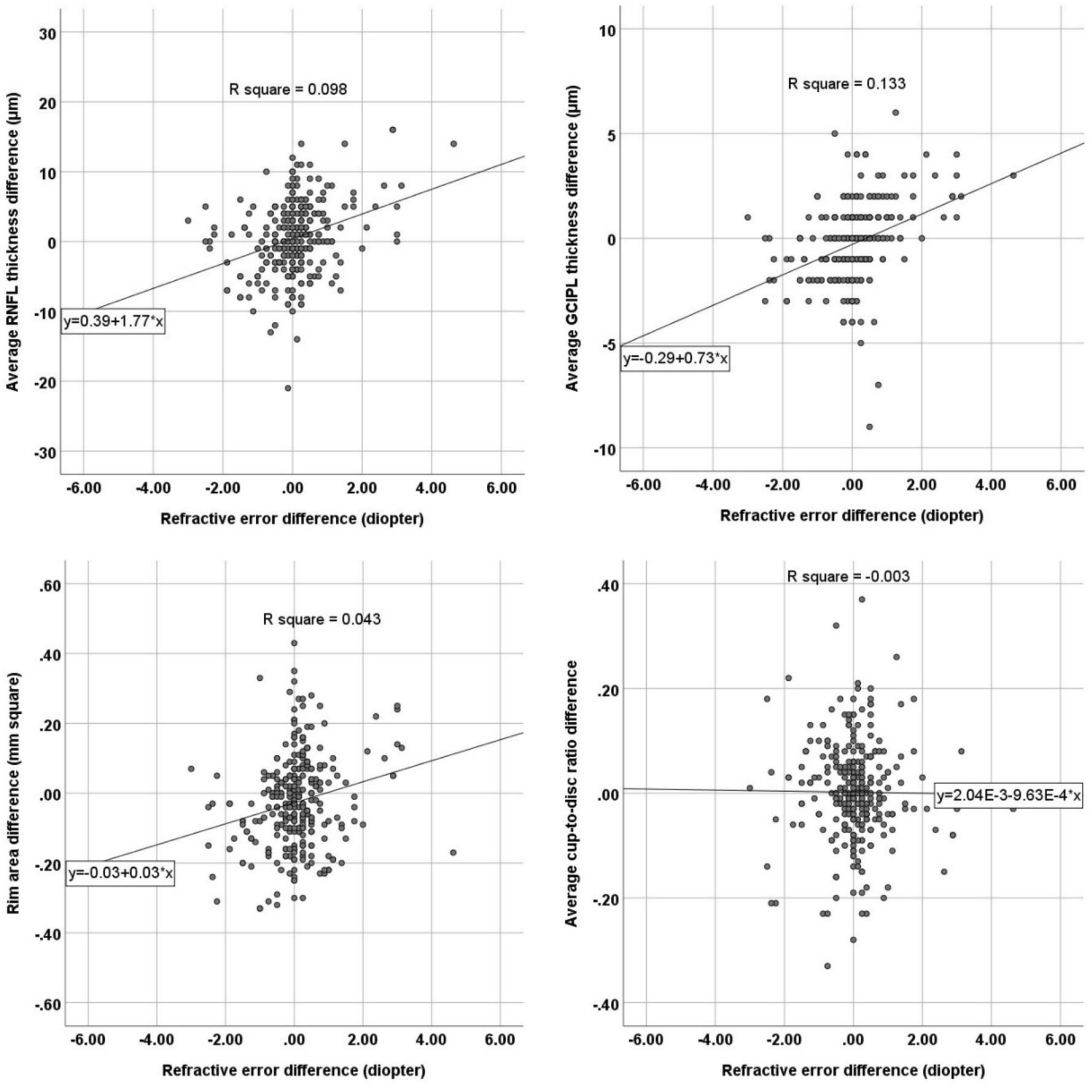
Scatter plot presenting the correlation between the interocular refractive error difference (right eye–left eye) and interocular differences in the average retinal nerve fiber layer (RNFL) thickness, average ganglion cell-inner plexiform layer (GCIPL) thickness, rim area, and cup-to-disc ratio measured by optical coherence tomography.

The age was not significantly associated with interocular differences in the OCT parameters (P > 0.05). Interocular differences in the refractive error were significantly associated with interocular differences in the average RNFL thickness (P < 0.001), average GCIPL thickness (P < 0.001), disc area (P = 0.043), and rim area (P < 0.001, Table 3, Fig. 2). However, the average cup-to-disc ratio (P = 0.847), vertical cup-to-disc ratio (P = 0.538), and cup volume (P = 0.776) were not significantly associated with interocular differences in the refractive error. Interocular differences in the disc size were significantly associated with interocular differences in the rim area (P < 0.001), average cup-to-disc ratio (P < 0.001), vertical cup-to-disc ratio (P < 0.001), and cup volume (P < 0.001, Table 3). No significant difference was found in any of the OCT parameters when the interocular difference was compared between females and males (P > 0.05). In the multivariate analysis, both interocular differences in refractive error (standardized beta = 0.157) and disc size (standardized beta = 0.456) were significantly associated with interocular differences in the rim area (P < 0.001, R^2^ = 0.249).

**Table 3:** Effect of age, interocular difference (right eye–left eye) in refractive error and disc size on interocular differences in optical coherence tomography parameters.

	Age		Refractive error		Disc size	
	Standardized beta	P value*	Standardized beta	P value*	Standardized beta	P value*
Average RNFL thickness	0.022	0.685	0.314	< 0.001	− 0.109	0.057
Average GCIPL thickness	0.012	0.828	0.366	< 0.001	0.065	0.254
Rim area	− 0.020	0.716	0.206	< 0.001	0.474	< 0.001
Average cup-to-disc ratio	− 0.027	0.637	− 0.011	0.847	0.627	< 0.001
Vertical cup-to-disc ratio	− 0.033	0.566	− 0.035	0.538	0.584	< 0.001
Cup volume	− 0.004	0.943	− 0.016	0.776	0.576	< 0.001

*RNFL* retinal nerve fiber layer, *GCIPL* ganglion cell-inner plexiform layer.

*Linear regression analysis.

## Discussion

In the present study, interocular differences were found in RNFL thickness, ONH parameters, and GCIPL thickness in healthy children and adolescents, except for in the cup-to-disc ratio and cup volume. The 2.5th and 97.5th percentile interocular difference tolerance limits were − 9.0 μm and 11.0 μm for the average RNFL thickness, − 0.21 and 0.18 for average cup-to-disc ratio, and − 4.0 μm and 4.0 μm for average GCIPL thickness, respectively. Interocular differences in RNFL and GCIPL thicknesses were affected by interocular differences in the refractive error. To the best of our knowledge, this is the first study to investigate the interocular asymmetry in the RNFL thickness, ONH parameters, and GCIPL thickness simultaneously in children and adolescents.

The interocular RNFL thickness symmetry in children and adolescents has been previously evaluated by several studies using spectral-domain OCT^4–8^. The 2.5th and 97.5th percentile interocular difference tolerance limits for average RNFL thickness were − 12.1 to − 9.0 μm for the 2.5th percentile and 8.9–13.0 μm for the 97.5th percentile^4–7^ which were similar to the present study results. Although the statistical significance of the interocular RNFL thickness differences has varied among studies, common findings were reported; the right eye had a thinner RNFL in the superior quadrant and a thicker RNFL in the temporal quadrant than the left eye^4–8^. Previous studies found that higher levels of myopia were associated with a thinner RNFL in the superior quadrant and a thicker RNFL in the temporal quadrant^15,16^. In our previous study evaluating the interocular symmetry of the RNFL thickness, the right eyes had higher myopia than the left eyes^17^. Therefore, we had speculated that the interocular RNFL thickness differences in the superior and temporal quadrants may be owing, in part, to the interocular refractive error differences^17^. However, in the present study and previous studies with children and adolescents, the interocular refractive error difference was not significant^4–8^. Therefore, other factors, including the interocular variation in topographic retinal blood vessel distributions^18^ or RGC axon and glial cell densities^19^ may contribute to the observed interocular asymmetry in the RNFL thickness.

When the ONH parameters were compared, the right eyes had lesser rim and disc areas compared to the left eyes. Previous studies reported no significant difference in ONH parameters^4,5,9^ or a greater vertical cup-to-disc ratio in the right eye compared to the left eye in children^7^. These discrepancies among the studies may be explained by the differences in study population characteristics. The 2.5th and 97.5th percentile interocular difference tolerance limits for the average cup-to-disc ratio were − 0.21 and 0.18, respectively. In previous studies, the 2.5th percentile ranged from − 0.11 to − 0.31, and the 97.5th percentile ranged from 0.14 to 0.25 for the average cup-to-disc ratio, respectively^4,7^.

Regarding the interocular symmetry of the macular RGC thickness in children, a study using Topcon 3D OCT-2000 (Topcon Corporation, Tokyo, Japan) reported that the right eyes had a thinner GCIPL in the superior hemisphere compared to the left eyes (mean difference, − 0.60 µm; P = 0.010), whereas in the inferior hemisphere (mean difference, 0.05 µm; P = 0.693) and total area (mean difference, − 0.09 µm; P = 0.111), the differences were not significant^10^. The 2.5th and 97.5th percentile interocular difference tolerance limits were − 2.5 μm and 2.0 μm, respectively for the total area GCIPL thickness^10^. These results are in line with the present study results. In the present study, the 2.5th and 97.5th percentile interocular difference tolerance limits were − 4.0 μm and 4.0 μm, respectively for the average GCIPL thickness and right eyes had significantly thinner average, superior, and superonasal GCIPL than the left eyes. Given that RNFL in the superior area corresponds to the superior RGC, this finding may be correlated with a thinner RNFL in the superior quadrant of the right eye than in the left eye. However, in another study investigating the interocular symmetry of the RNFL and GCIPL thickness in adults, although the right eyes had thinner superior RNFL and thicker temporal RNFL, no significant difference was found in the GCIPL thickness^1^. To date, this is the only study reporting the interocular symmetry of the GCIPL thickness in children and adolescents using Cirrus HD-OCT. Further studies with various populations are needed to validate our results.

The age and gender did not affect the interocular symmetry in the RNFL thickness and ONH parameters, which agrees with the results of previous studies^4–10^. Given that OCT parameters change with age^20,21^, further studies assessing longitudinal changes in the OCT parameter symmetry are needed.

In the present study, an eye with a greater interocular asymmetry in the refractive error had a greater interocular asymmetry in the RNFL thickness, disc area, rim area, and GCIPL thickness. It has been reported that an eye with higher myopia has a thinner RNFL^15,16^ and GCIPL^22,23^. This finding may contribute to the effect of the refractive error on OCT parameters. Previous studies also reported that the interocular GCIPL thickness difference was significantly associated with the interocular refractive error^10^ or axial length difference^1^. In contrast, the average cup-to-disc ratio, vertical cup-to-disc ratio, and cup volume symmetry were not significantly affected by the refractive error symmetry. Given that the interocular differences in these parameters were not significant, this finding may be expected. We suggest that the cup-to-disc ratio and cup volume may be useful for the detection of glaucoma or other optic neuropathies based on interocular comparison, especially in cases with an asymmetric refractive error. Further investigation is required to address this issue.

Many OCT studies have included one eye of an individual selected randomly or by disease severity. However, the laterality of the included eyes may have affected the OCT results, especially the RNFL and GCIPL thicknesses of the superior area. Therefore, when comparing OCT parameters between groups, different distributions of laterality may affect the study results.

In the present study, a cup-to-disc ratio of > 0.5 or an asymmetry of > 0.2 were considered as exclusion criteria to exclude individuals with glaucoma. However, there may have been individuals with eyes that have a cup-to-disc ratio > 0.5 or an asymmetry of > 0.2 without glaucoma. Therefore, the inclusion of only individuals without this condition may have caused a selection bias. Given that glaucoma without high intraocular pressure in children or adolescents is rare, further studies including all individuals, regardless of the cup-to-disc ratio without high intraocular pressure, are needed.

Only Korean participants were included in the current study. The use of a single ethnicity minimizes the confounding effects of ethnicity. However, this may be a limitation of the present study in terms of the generalizability of the results. Axial length is an important factor affecting the OCT parameters^15,24^. However, in the present study population, axial length was not measured routinely. Therefore, the effect of axial length on OCT parameters could not be analyzed. Further studies including populations with various ethnicities and data regarding axial length may be needed.

In conclusion, interocular differences were found in the RNFL thickness, ONH parameters, and GCIPL thickness in healthy children and adolescents. However, cup-to-disc ratio and cup volume did not show significant interocular differences and were less likely to be affected by the refractive error. These findings should be considered when interpreting the OCT measurements.

## Materials and methods

### Participants

This retrospective cross-sectional study protocol was approved with the waiver of informed consent by the Institutional Review Board of Kim’s Eye Hospital, Seoul, Republic of Korea, and all study procedures adhered to the tenets of the Declaration of Helsinki. Individuals who visited Kim’s Eye Hospital, aged between 5 and 17 years, for a regular health examination were enrolled. Each participant underwent a full ophthalmic examination, including the assessment of the visual acuity, refractive error by cycloplegic refraction, and anterior segment using slit-lamp biomicroscopy and fundus examination with a 90 diopter (D) lens. In addition, all participants underwent OCT examination using Cirrus high-definition spectral-domain OCT (Cirrus HD-OCT; Carl Zeiss Meditec, Dublin, CA, USA) to evaluate the circumpapillary RNFL thickness, ONH parameters, and macular RGC thickness.

The inclusion criteria included the presence of a best-corrected visual acuity of 20/30 or better, normal anterior segment, normal ONH with no glaucomatous changes (that is, large cup-to-disc ratio, neuroretinal rim narrowing, or disc hemorrhage), and a normal retina. Individuals with strabismus, amblyopia, a cup-to-disc ratio > 0.5, asymmetry of > 0.2 between fellow eyes, a history of previous ocular surgery, and a history of prematurity, developmental abnormality, or neurological or systemic diseases were excluded.

### OCT measurements

A 200 × 200 cube optic disc scan and macula scan were obtained using Cirrus HD-OCT with eye tracking. To acquire the images, the scanning laser was focused after the participants were seated and properly positioned within the chin rest. Using the iris and fundus views, the ONH or macula was aligned so that it presented on the center of the scan. Once the ONH or macula was centered on the live scanning laser image, data from a 6 × 6 mm area were captured. The Cirrus HD-OCT algorithm automatically determines the vitreoretinal surface and posterior boundary of the RNFL and presents the circumpapillary RNFL thickness values in the global area (average) and four quadrants (superior, nasal, inferior, and temporal). Various ONH parameters, including the rim area, disc area, average cup-to-disc ratio, vertical cup-to-disc ratio, and cup volume, are automatically generated by the Cirrus HD-OCT algorithm. Cirrus HD-OCT automatically segments the anterior border of the inner plexiform layer and posterior border of the RGC and shows average, minimum, and sector-based (superotemporal, superior, superonasal, inferonasal, inferior, and inferotemporal) macular ganglion cell-inner plexiform layer (GCIPL) thickness values. Only images without prominent involuntary saccade artifacts and a signal strength ≥ 7 were included in the analyses.

### Statistical analyses

A paired t-test was performed to evaluate the significance of interocular differences (right eye–left eye) in a refractive error presented as the spherical equivalent and OCT parameters. The relationship between the age, interocular refractive error differences, interocular optic disc size differences, and interocular OCT parameter differences were evaluated using linear regression analysis. The interocular difference of variables was compared between female and male participants using an independent t-test. Among the independent variables, those with significant association in univariate analysis (P < 0.05) were included in the multivariate analysis. A commercial software (SPSS version 12.0; SPSS, Chicago, IL) was used to perform all statistical analyses, and statistical significance was defined as P < 0.05.

## Data Availability

The datasets used and/or analyzed during the current study are available from the corresponding author on reasonable request.
